# Sire Breed, Litter Size, and Environment Influence Genetic Potential for Lamb Growth When Using Sire Breeding Values

**DOI:** 10.3390/ani12040501

**Published:** 2022-02-17

**Authors:** Khama R. Kelman, Clair Alston-Knox, David W. Pethick, Graham E. Gardner

**Affiliations:** 1 Australian Cooperative Research Centre for Sheep Industry Innovation, Armidale, NSW 2351, Australia; d.pethick@murdoch.edu.au (D.W.P.); g.gardner@murdoch.edu.au (G.E.G.); 2 Centre for Animal Production and Health, College of Science, Health, Engineering and Education, Murdoch University, Murdoch, WA 6150, Australia; 3 Predictive Analytics Group, Melbourne, VIC 3000, Australia; calston-knox@pa-group.com.au

**Keywords:** lamb, growth curve, regression, growth rate, breeding value

## Abstract

**Simple Summary:**

Previous studies have demonstrated a reduced response to sire growth breeding values when there is poor ewe nutrition, resulting in lighter lambs. The current study has demonstrated that production factors which influence lamb growth, such as birth type, rear type, or sire breed, have a similar effect. The reduced growth in lambs is quantified in this study to provide lamb producers with realistic expectations of lamb growth, when high growth sires are used in combination with production factors which influence growth.

**Abstract:**

Lamb growth can be optimised with genetic selection using sire Australian sheep breeding values, however, breeding value expression has been shown to be reduced with poor nutrition. It was therefore hypothesised that the genetic potential for lamb growth would also be reduced, where production factors such as multiple births limit growth. Live weights at birth, weaning, and post-weaning were collected from more than 18,000 lambs produced over five years and eight locations of the Sheep Cooperative Research Centre Information Nucleus Flock experiment, and the impact of environment, production factors, and genotype was determined using mixed effects regression. The genetic potential for lamb growth was moderated by environment, multiple births, and sire type (*p* < 0.05). Twin lambs achieved 76% of the expected weight gain at weaning and 58% post-weaning. For triplet lambs weight gains were drastically less at approximately 30% of the expected gain at the same time points. Lambs born to maternal sires consistently had the poorest response to genetic selection, achieving approximately half the expected weight gain. Hence, producers need to temper expectations for growth based on genetic selection, or employ mitigation strategies such as precision feeding, the use of alternate breeds, or place emphasis on the genetic merit of other desirable traits.

## 1. Introduction

Weighing lambs at key time points in production is a common practice employed among sheep enterprises. Lamb weights can be used to inform precision feeding or management strategies; for example, to reach target slaughter weights, predict puberty [1], or optimise carcass traits to improve eating quality [2]. Lamb weights are known to be influenced by production factors such as multiple births, dam age, and the sex of the lamb, with ewe lambs, lambs from 1 year old dams, and multiple birth lambs tending to be lighter at birth [3]. Environmental factors such as the site and timing of birth also have an effect, with lamb weight increasing by up to 10% between seasons for lambs born at the same site [3]. Lamb genotype has a large influence on lamb weight, with lambs born to terminal sires being generally heavier than those born to maternal or Merino sires [4]. Therefore, producer expectations of lamb weight need to be tempered by environment and where the enterprise structure or breeding objectives favor specific genotypes or production factors that can influence lamb weight, such as increased litter size in prolific flocks.

Lamb growth is also influenced by genetics, with producers using Australian sheep breeding values (ASBVs) to select sires for increased growth via an increased sire weaning weight (WWT_BV_) and post-weaning weight (PWT_BV_). Selection for increased post-weaning weight has been shown to correlate with an increased mature size [5], however, the full expression of the sire breeding value can be reduced by poor ewe nutrition [6], resulting in restricted growth. The interaction of sire ASBVs for different stages of growth with environmental, production, or genotypic factors that influence growth has not been fully evaluated in lambs with different genetic potentials for growth.

The aims of this study were threefold. First, to establish the range of live weights and growth rates achievable by lambs of modern genotypes under commercial conditions. Second, to demonstrate and quantify the effect of environmental, production, and genotypic factors that influence lamb growth. Third, to determine whether these factors limit the full expression of sire ASBVs in lambs born to high growth sires. This will provide producers with realistic expectations of growth to enhance trust, and therefore increase the likelihood of using breeding values. We hypothesized that factors known to influence growth, such as multiple births, would limit the full expression of sire ASBVs for growth, preventing lambs born to high growth sires from achieving their predicted genetic outcomes.

## 2. Materials and Methods

### 2.1. Experimental Design

The design of the Sheep Cooperative Research Centre Information Nucleus Flock was presented in detail by Fogarty et al. [7] and van der Werf et al. [8]. Each year for 5 years, approximately 3500 lambs were produced by artificial insemination, using the same key industry sires at each of the eight commercial-like research site locations across Australia (Kirby NSW, Trangie NSW, Cowra NSW, Rutherglen VIC, Hamilton VIC, Struan SA, Turretfield SA, and Katanning WA). The sites represent a broad range of production systems across wheat–sheep and high rainfall zones [9]. The lambs were the progeny of 435 key industry sires, including maternal sires (Border Leicester, Bond, Booroola, Coopworth, Corriedale, Dohne Merino, East Friesian, Prime SAMM, and White Dorper), Merino sires (Merino and Poll Merino), and terminal sires (Hampshire Down, Ile De France, Poll Dorset, Southdown, Suffolk, Texel, and White Suffolk). Maternal and Merino sires were mated to Merino ewes, while terminal sires were mated to either Merino or commercial maternal (Border Leicester-Merino) ewes. Lambs were raised under commercial pasture grazing conditions and supplemented with grain, hay, or feedlot pellets when feed supply was limited [10]. Lambs were born in winter and weighed at birth, weaning, and approximately every two weeks thereafter.

### 2.2. Data Available

A total of 17,525 lambs with 142,572 weight recordings were available. The weight and growth rate ranges are presented in Table 1.

Of the 435 sires used, 84 maternal, 167 Merino and 184 terminal had sire ASBVs available. The breeding values for weight were based on live weights adjusted to a constant age, while breeding values for post-weaning fat depth (PFAT_BV_) and post-weaning eye muscle depth (PEMD_BV_) were based on live ultrasound measurements at the c-site (45 mm from the midline over the 12th rib). All sire ASBVs were sourced from Sheep Genetics [11], where values were generated within three separate data bases for terminal, maternal and Merino sires. In all cases, the databases used to calculate the sire breeding values did not include the progeny analysed in this experiment. A correlation matrix of these sire ASBVs is presented in Table 2.

### 2.3. Statistical Analysis

Three separate stages of analysis occurred. Firstly, a growth curve was fitted to each individual lamb’s live weight data, then weights and growth rates at key time points were estimated based on the curve. Secondly, these estimates were then analysed as dependent variables to determine the environmental, production, and genotypic factors which influence the estimates. These are referred to as the base models. Finally, sire ASBVs generated independently by Sheep Genetics were included as covariates in the base models from the second stage, in order to determine the impact of the sire’s ASBV on progeny weight and growth rate estimates, and to determine whether environmental and production factors that influence growth also impacted the phenotypic expression of the ASBV effect.

More extensively, for stage one of the analysis, weights and growth rates were estimated for each animal at three industry-relevant time points. These were day 100 to represent weaning age, day 150 to represent the post-weaning time point, and day 240 representing near the average age at which PWT_BV_ is calculated in the Sheep Genetics database [11]. Lamb weights and growth rates were modelled using a random effects model, as this methodology could account for lambs with as few as one weight recording, and not every lamb was weighed at the specific time points of interest. Analysis was completed using the MCMCglmm library [12] in the R package [13]. Models were developed to describe live weight, which included fixed environmental effects for research site locations (Kirby, Trangie, Cowra, Rutherglen, Hamilton, Struan, Turretfield, and Katanning) and year of birth (2007, 2008, 2009, 2010, and 2011), fixed production effects for sex (male and female), birth-type–rear-type combination (single–single, twin–single, twin–twin, triplet–single, triplet–twin, and triplet–triplet) and age of the dam (2, 3, 4, 5, 6, 7 and 8 years), and fixed genotypic effects for sire type (Merino, maternal, and terminal) and dam breed within sire type (Merino × Merino, maternal × Merino, terminal × Merino, terminal × Border Leicester-Merino). Individual identification was included as a random factor, as individual progeny were weighed at multiple time points. Similarly, sire identification and dam identification by year were included as random factors, as sires were represented by multiple progeny, and dams were represented by multiple progeny across multiple years. All factors, including their interactions with animal age, were modelled using linear, quadratic, and cubic terms for time (age in days).

For stage two of the analysis, the weights and growth rates estimated in stage one were then analysed in SAS using a linear mixed-effects model (SAS Version 9.1, SAS Institute, Cary, NC, USA), where fixed and random effects were tested using likelihood ratios. These base models (including actual birth weight, estimated weights at 100, 150, and 240 days, and estimated growth rates at 100, 150, and 240 days) contained the same fixed effects as described in the random effects model above, however, these effects did not vary with time.

For stage three, sire growth ASBVs were included in the relevant base models. To assess the impact of the sire birth weight ASBV (BWT_BV_) on progeny weight at birth, BWT_BV_ was included as a covariate in the birth weight base model. The model contained the first-order interactions of production, environmental, and genotypic effects with BWT_BV_, and included a quadratic effect of the covariate. Similarly, to assess the impact of sire WWT_BV_ on progeny weight and growth rate at weaning (100 days), WWT_BV_ was included as a covariate in the 100-day base models. As producers use industry indices to select for growth, muscling, and leanness at the post-weaning time point (150 days), PWT_BV_, PEMD_BV_, and PFAT_BV_ were all included in the base model to assess their impact on weight and growth rates at 150 and 240 days. This enabled us to test the genetic propensity for reduced fatness and increased muscularity to impact phenotypic growth. All models contained relevant first-order interactions between fixed effects, which were removed in a stepwise manner by a process of backward elimination if non-significant (*p* < 0.05).

## 3. Results

### 3.1. The Effect of Enrivonment, Production Factors, and Genotype on Lamb Growth

Environmental factors (site and year of birth), production factors (sex, birth-type–rear-type, and dam age) and genotype (sire type and dam breed within sire type) all affected lamb weight (Table 3).

As environmental factors are not replicable, only weight extremes for site and year are presented. Lambs were generally the lightest on average in Kirby, weighing 3.90 (±0.04), 21.61 (±0.32), and 27.18 (±0.26) kg at birth, 100, and 150 days (*p* < 0.01). Conversely, lambs were generally the heaviest on average in Trangie, weighing 31.87 (±0.39), 40.38 (±0.32), and 53.61 (±0.17) kg at 100, 150, and 240 days (*p* < 0.01). Lambs born in 2011 were generally the heaviest on average, weighing 4.77 (±0.43), 29.16 (±0.43) and 35.18 (±0.35) kg at birth, 100, and 150 days (*p* < 0.01). In no year were lambs consistently the lightest. During early growth, the difference in average weight between years (the site by year interaction) was consistently the smallest in Cowra and the largest in Katanning. At the Katanning site, lamb weight varied between years by as much as 40% of the average lamb weight.

Production factors impacted lamb weight, with wether lambs born as singles to older dams having the largest weight advantage (Table 4). Wether lambs grew faster at all time points, and were between 5 and 8% heavier than ewe lambs. Single-born lambs were on average 1.02 kg heavier than twin lambs at birth, and dramatically heavier (1.74 kg) than triplets (*p* < 0.01). At weaning single-born lambs grew faster than twin- or triplet-born lambs (140.20 vs. 135.70 vs. 133.40 g/day). This effect reversed early post-weaning, and multiple-born lambs grew faster (90.53 vs. 104.00 vs. 114.30 g/day). Despite this compensatory growth, multiple-birth lambs were still lighter than single-born lambs at day 240. Twin- or triplet-born lambs that experienced sibling mortality were growing faster at weaning and were heavier from weaning onwards than multiple-birth lambs that did not experience sibling mortality (*p* < 0.01). Lambs born to mature (8 year old) dams were 10% heavier than lambs born to immature (2 year old) dams (*p* < 0.01), and were the fastest growing at weaning (162.50 vs. 118.20 g/day) and early post-weaning (156.60 vs. 58.48 g/day), continuing to be 10% heavier than lambs born to immature dams at day 240. Lamb birth weight increased substantially for each year that dam age increased between 2 and 4 years of age (0.22 kg/year), although the weight differential was inconsistent after birth.

Growth varied between genotypes, with terminal-sired lambs being heavier and faster-growing than the progeny of Merino sires (*p* < 0.01) (Table 4). At birth, lambs born to Merino sires were 11% lighter, and this difference increased to 42% at 240 days. Within the terminal-sired lambs, those from Border Leicester-Merino dams were between 10 and 16% heavier than those born to Merino dams.

The range of the effect was calculated as the difference between the smallest and largest predicted mean for each factor (Table 5). For birth weight, the difference between predicted mean weight for the single birth type (heaviest) and triplet birth type (lightest) was 1.74 kg, which was larger than for all other environments, production factors, or genotypes. Site had the largest potential impact on weight after birth, followed by sire type.

### 3.2. Breeding Value Effects on Lamb Growth

Sire as a random effect was significant (*p* < 0.01) in the base models used to assess the impact of breeding value effects on estimated weight and growth rate.

Regression coefficients for sire growth ASBVs, and how they varied with environment, production factors, and genotype, are presented in Table 6. On average, lamb birth weight increased by 0.53 kg/1 kg increase in BWT_BV_. The range in BWT_BV_ was 1.8 kg, and this equated to an increase in lamb weight of 0.95 kg at birth between lambs born to sires with the lowest and highest BWT_BV_ (Table 5). The effect per kg of sire ASBV was slightly less at weaning, with lamb weaning weight increasing by 0.37 kg/1 kg increase in WWT_BV_. The range in WWT_BV_ was 16 kg, equating to an increase in lamb weight of 5.98 kg. Lamb weight increased by 0.28 kg/1 kg increase in PWT_BV_ at 150 days and 0.36 kg/1 kg increase at 240 days. The range in PWT_BV_ was 23 kg, which equated to increases in lamb weight of 6.62 and 8.24 kg at 150 and 240 days, respectively. There was generally no association between growth ASBVs and growth rate.

Lamb growth was not affected by selection for PEMD_BV_, while selection for PFAT_BV_ was associated with a small increase in post-weaning weight at 150 days, equating to 1.41 kg across the 5 mm PFAT_BV_ range (*p* < 0.01).

### 3.3. Variation in Breeding Value Effects on Lamb Growth between Environment, Production Factors and Genotype

Sire ASBVs for growth varied between environment, production factors, and genotype, resulting in different coefficients for the regression (Table 6), indicating that these factors moderated the genetic potential for growth in progeny. The association between growth ASBVs and the environmental factors of site and year was inconsistent and varied with lamb age. More consistent effects were seen with birth type and sire breed.

The association between BWT_BV_ and actual lamb birth weight varied with the year of birth and birth type (*p* < 0.01). Between birth types, lamb weight increased by as much as 0.70 kg/1 kg increase in BWT_BV_ in single-born lambs, and as little as 0.37 kg in triplet-born lambs. This pattern remained consistent at weaning and post-weaning, where the coefficient diminished with multiple births and number of siblings, indicating that these lambs did not achieve the expected growth outcomes based on selection for growth using sire ASBVs.

**Figure 1:**
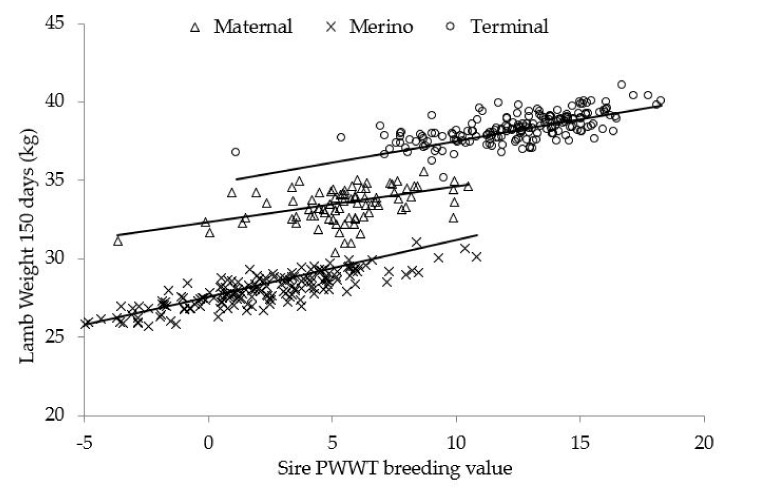
Comparison of sire estimates for post-weaning weight (PWT_BV_) Australian sheep breeding values for maternal, Merino, and terminal sires, with the predicted means for lamb weight at 150 days. Lines represent the predicted means for weight for each sire type. Symbols (∆, X, O) represent individual sire estimates.

Expected growth outcomes when selecting for growth using sire ASBVs varied between sire types, with the progeny of Merino sires consistently having higher coefficients than the progeny of terminal or maternal sires. Lamb weight increased by 0.55 kg per kg increase in sire WWT_BV_ at 100 days for Merino, 0.29 kg for terminal, and 0.28 kg for maternal sire types. This equated to an increase in lamb weight of 6.66, 2.91, and 2.31 kg across the respective 12.11, 10.04, and 8.26 kg WWT_BV_ ranges. For each kg increase in sire PWT_BV_ at 150 days, lamb weight increased by 0.35 kg for Merino, 0.29 kg for terminal, and 0.22 kg for maternal sire types. This equated to an increase in lamb weight of 5.54, 4.98, and 3.11 kg across the 15.83, 17.16, and 14.14 kg PWT_BV_ ranges (Figure 1). At 240 days, the association between PWT_BV_ and lamb weight varied between years and dam ages (*p* < 0.01), although the impact was small.

There were no main associations between sire ASBVs for growth and lamb growth rate. A weak association was present between PWT_BV_ and growth rate at 240 days within some birth-type–rear-type combinations, varying by as much as 3.99 g/day in triplet-born and -raised lambs, and as little as −2.14 g/day in single-born lambs. There was an association between PEMD_BV_ and weight at 240 days, although the effect was only present at the Katanning site, where weight reduced by 0.12 kg per kg of PEMD_BV_, equating to a reduction of 0.96 kg across the 8 kg PEMD_BV_ range.

## 4. Discussion

### 4.1. Association between Breeding Values and Lamb Growth

As expected, the progeny of sires with increased BWT_BV_, WWT_BV_, and PWT_BV_ were heavier at birth, weaning, and post-weaning, respectively. However, expected growth outcomes were shown to vary, and sometimes be suppressed, between sites, years, birth types, and sire types.

Sites with the largest lambs (Trangie and Cowra) also had weight responses closest to what is expected with selection for growth using sire ASBVs; meanwhile, sites with the lightest lambs (Kirby) had smaller than expected responses. Nutrition varied between sites, and is known to moderate breeding value effects [6], so this may be a contributing factor to these findings, although other factors such as the difference in dam genetics between sites cannot be discounted. Precision feeding would optimize resource allocation and may minimise the effects of site that are caused by nutrition. Interestingly, this effect was not present at birth, which may indicate a buffering of the lambs by maternal nutrition, with dams mobilising more of their own energy reserves. Alternatively, variation may have been present but obscured by the fact that the error represents a larger proportion of the difference between weights at birth than later in life. At 240 days, the expression of sire PWT_BV_ did not differ between sites. It is possible that at this point in time, the extremes for the sites with the poorest nutrition were not enough to reduce the response to PWT_BV_.

Lamb birth-type and rear-type had a large impact on the weight responses of lambs selected for growth using sire ASBVs, with this study identifying the range of the effect. Single-birth lambs had the greatest expression of their genetic potential for growth, making this a highly profitable selection trait in flocks with a lower occurrence of multiples. Multiple-birth lambs had consistently poorer responses to sire ASBVs for growth, and producers with prolific flocks need to temper their expectations of growth as litter size increases, or focus on sires with genetic merit for other desirable traits. The suppression of growth potential with multiple births was partially alleviated by sibling mortality. For example, at weaning, the weight of twins born and raised as twins increased by 0.38 kg per kg increase in WWT_BV_, while the response was greater in twin-born lambs raised as singles, increasing by 0.54 kg. The effect on growth rate was moderated at 240 days, where the response to increased sire PWT_BV_ was positive in lambs born and raised as triplets, and in contrast was negative in lambs born as singles. This is likely due to compensatory growth being experienced by triplets when nutritional restriction is lifted [14] post-weaning. Dakhlan et al. [15] also demonstrated an interaction between genetic potential for growth and birth type when studying Merino lambs. In comparison to this study, the effect was present only at birth and weaning.

Weight responses of lambs selected for growth were sensitive to sire type, with the progeny of Merino sires having the greatest response. As Merino-sired progeny have lower growth rates and a smaller mature size [16], they are likely to be proportionately less of their mature size at weaning and post-weaning, and therefore have an increased expression of sire ASBVs for growth. Sire ASBVs are calculated as for single-born lambs at the given age (e.g., birth for BWT_BV_). As sires contribute half of the lamb’s genetics, lamb weight is expected to increase by 0.5 kg for each 1 kg increase in sire growth ASBV. While this was seen in the progeny of Merino sires, the progeny of maternal and terminal sires had a much lower response, being only 60% of the expected weight increase. Maternal sires had a reduced accuracy of sire ASBVs compared to Merino sires, which may account for some of the variation in sire-type performances.

Although lambs from Border Leicester-Merino dams were larger and grew faster than lambs from Merino dams, there was no differential expression of the genetic potential when selected for growth, so growth expectations by Mernio or maternal breeders need not vary. This is despite evidence of reduced milk yield from Merino dams [17], and lower growth rates at weaning compared to the progeny of Border Leicester-Merino dams. It is not clear why there was no breeding value differential, however, the growth restriction of lambs due to dam breed may not be enough to reach a threshold beyond which there is an impact on the expression of breeding values. Overall, the range of the production effects of site, birth-type–rear-type combinations, and sire type were between 1.5 and 3.5 times the range of the effect of dam breed on weight. Production effects which vary with WWT_BV_ and PWT_BV_ follow a similar pattern. As dam breed only had a small impact on lamb weight, any variation in the expression of the breeding value may have been too small to detect, despite a wide range in sire ASBVs values.

Selection for increased muscle via an increased sire PEMD_BV_ did not impact on progeny weight. Similarly, Gardner et al. [18] demonstrated no association between PEMD_BV_ and pre-slaughter live weight. As breeding values are calculated corrected to a constant live weight [11], their impact on the phenotypic expression of growth is limited. Selection for leanness via a reduced PFAT_BV_ did increase lamb weight although the effect was small, representing an increase of 4% above the average lamb weight, and only present at 150 days. Gardner et al. [18] showed a similar association with pre-slaughter live weight being around 10% higher than the average lamb weight. The phenotypic correlation between c-site ultrasound fat depth and weight was relatively small in the Huisman and Brown [5] study, which may reflect the lack of a consistent effect on weight in this study.

### 4.2. Association between Environment, Production Factors, Genotype, and Lamb Growth

The site at which lambs were reared impacted weight throughout life, with lambs at Trangie being consistently heavier and lambs at Kirby being consistently lighter. These differences are likely to be partly explained by site nutrition [19] and dam genetics. If dam genetics at each site were entirely responsible for the differences in lamb weight, there would be minimal differences in lamb weights at each site across different years; however, lamb weight at a single site varied between the years by nearly 40% of the average lamb weight. Thus, while dam genetics varies between sites and contributes to lamb weight differences, they are not solely responsible for these differences. Quantifying the variations within a site, between years, provides producers with a realistic expectation of how lamb growth can vary, allowing them to make responsive precision management decisions about joining, feeding, stocking rate, and target slaughter dates.

The effect of year of birth on lamb weight was not as consistent, having no impact on birth weight, and a small impact between 100 and 240 days. The effect was as small as 11% of the range of site of rearing, which had the largest effect (Table 5). This effect may be due to post-weaning nutritional availability within a year which would explain why the impact is not present at birth, as well as non-nutritional factors that contributed to the impact of lamb weight within a year.

Male lambs were heavier and grew faster at all times measured, and this has been demonstrated in previous studies [3,20,21]. Despite male lambs being castrated in this study, this effect is consistent with the impact of testosterone, a potent muscle growth stimulant [22]. Thatcher, Warren, and Nicholls [21] demonstrated significantly faster growth in rams compared to wethers (*p* < 0.05), thus the differential in growth between wethers and ewe lambs in this study is likely to have been further increased if the male lambs were left entire. Producers can utilise these growth differentials to predict puberty in ewe lambs or slaughter dates in wether lambs based on the focus of the enterprise. Single-born lambs were 22% heavier than twin-born lambs and 45% heavier than triplet-born lambs at birth, which is consistent with the findings of Afolayan, Fogarty, Ingham, Gilmour, Gaunt, Cummins, and Pollard [3] and with Greenwood, Slepetis, and Bell [23], who demonstrated placentally mediated foetal growth restrictions with multiple births. Weight differences due to birth type were present throughout life, although they were proportionately smaller with increasing age. Between birth and weaning, milk production fuels lambs and is highly correlated with lamb growth [24,25]. However, milk yield varies little between single- and twin-bearing ewes, with daily yield bring only 14% higher and peak yield only 27% higher in twin-bearing ewes, despite mild yield doubling in peak lactation for both groups [26]. This represents a nutritional restriction between birth and weaning for lambs raised as twins and limits their potential for growth. Within birth-type, weight also varied with rear-type, with multiples raised as singles being heavier than lambs raised as multiples. These weight differences were also present throughout life, although they reduced after weaning, when triplet-born lambs raised as singles had the greatest difference, being 26% heavier than triplet-born lambs raised as triplets.

Hence, both birth-type and rear-type impact on lamb weight, with weight differences at birth likely caused in part by the effects of in utero and pre-weaning nutritional restriction with multiples. At 100 days, the triplet-born and -raised lambs had the slowest growth rate; however, by 150 days, their growth rate was the fastest, suggesting that multiple-born lambs experience a period of compensatory growth once nutritional restriction is lifted [14] at weaning. Producers selecting for more prolific flocks may need to employ mitigation strategies such as foetal ageing and differential feeding to optimize lamb survival and growth. Dam age influenced lamb birth weight, with lambs born to 2 year old ewes, the youngest in this experiment, being the lightest. This may be due to the utilisation of energy by the 2 year old dam for its own growth and development at the expense of the lamb, causing in utero nutritional restriction. Ali et al. [27] demonstrated a small increase in birth weight of 25 g for each one-year increase in dam age for Rambouilet ewes, until dams reached 4 years of age. This was far smaller than the 230–430 g increase between years seen in this study, and may be a reflection of the variation in the dam age effect between dam breeds. Afolayan, Fogarty, Ingham, Gilmour, Gaunt, Cummins, and Pollard [3] used crossbred ewes, and the increase in lamb weight at birth between dam years was as much as 630 g. While there was no dam age by dam breed effect in this study, Rambouilet ewes were not used, and the dam breeds which were used may not have been divergent enough to drive an effect. Dam age also influenced weaning and post-weaning lamb weight, with lambs from 4–6 year old dams tending to be heavier at 100 and 150 days. Increases in lamb weight at birth, weaning, and post-weaning (200 days) with increasing dam age were also found in the Afolayan, Fogarty, Ingham, Gilmour, Gaunt, Cummins, and Pollard [3] study, although only 1–4 year old dams were included, so the effect beyond this age is unknown. At 240 days, when lambs were close to adult weight, this pattern was no longer evident in this study, and the difference between lambs born to ewes three years or older was less than 1 kg. Therefore, culling ewes for age is not substantiated based on lamb weight alone. It is possible that the early influence of dam age on lamb weight is due in part to factors such as mothering ability, milk production, and colostrum composition, the effects of which diminish post-weaning. However, these effects were not measured during this experiment.

The progeny of terminal sires were heavier between birth and 240 days, while the progeny of Merino sires were lighter and had the slowest growth rates, which is consistent with the findings of Fogarty, Hopkins, and van de Ven [4]. Furthermore, the progeny of elite terminal sires grew in excess of 250 g/day from weaning. As many nutritional requirement tables used for ration formulation only accommodate lamb growth rates up to 250 g/day [28], the nutrient requirements for the progeny of contemporary elite growth sires need to be updated. Within the progeny of terminal sires, those from Border Leicester-Merino dams were heavier and had higher growth rates than those from Merino dams, which is a reflection of the increased mothering ability [29] and milk yield of Border Leicester-Merino dams [17].

## 5. Conclusions

Lamb growth is influenced by on-farm production factors and genetic selection for growth using sire ASBVs. The production factors of site, birth-type–rear-type combinations, and sire type had the greatest impact on lamb growth, with single-born wether lambs from terminal sires being heavier at all key time points. The range of their impact on growth was as much as twice the range of genetic selection using sire ASBVs. The genetic potential of lambs selected for growth may not be realised when combined with other factors that influence growth, such as sire type, and lamb birth-type and birth environment. Producers may temper their expectations of growth, or employ mitigation strategies such as precision feeding, the use of alternate breeds, or place emphasis on the genetic merit of other desirable traits.

## Figures and Tables

**Table 1:** Descriptive statistics for actual lamb birth weight (kg), estimated weights (kg), and growth rates (g/day) at 100, 150, and 240 days of age.

Growth Measurement	Mean	Minimum	Maximum	Range	Standard Deviation
Birth weight (kg)	4.91	1.40	10.10	8.70	1.06
Weight 100 days (kg)	28.70	3.38	60.76	57.38	8.17
Weight 150 days (kg)	34.38	10.81	68.78	57.97	7.84
Weight 240 days (kg)	43.42	21.24	74.94	53.70	8.90
Growth rate 100 days (g/day)	136.96	−184.43	547.70	732.13	80.52
Growth rate 150 days (g/day)	97.17	−170.23	393.49	563.72	67.23
Growth rate 240 days (g/day)	125.11	−559.42	897.38	1456.80	167.58

**Table 2:** Simple correlations (standard errors) of Australian sheep breeding values for sires’ post-weaning weight (PWT_BV_), fat depth (PFAT_BV_), and eye muscle depth (PEMD_BV_), among maternal, Merino and terminal sires.

	PWT_BV_	PFAT_BV_	PEMD_BV_
	Maternal		
PWT_BV_	1.00	−0.18 (0.09)	−0.06 (0.62)
PFAT_BV_		1.00	0.47 (0.00)
PEMD_BV_			1.00
	Merino		
PWT_BV_	1.00	0.23 (0.00)	0.29 (0.00)
PFAT_BV_		1.00	0.76 (0.00)
PEMD_BV_			1.00
	Terminal		
PWT_BV_	1.00	−0.03 (0.69)	−0.26 (0.00)
PFAT_BV_		1.00	0.39 (0.00)
PEMD_BV_			1.00

**Table 3:** Base models, including numerator and denominator degrees of freedom and F-values, of the impact of environment, production factors, and genotype on actual lamb birth weight and estimated weights and growth rates at 100, 150, and 240 days of age. Asterisks represent significant effects within each model (column).

	Birth Weight		Day 100			Day 150			Day 240		
				Weight	Growth Rate		Weight	Growth Rate		Weight	Growth Rate
	NDF ^1^; DDF ^2^	F-value	NDF; DDF	F-value	F-value	NDF; DDF	F-value	F-value	NDF; DDF	F-value	F-value
Site	7; 5085	473.89 **	7; 5099	778.80 **	306.43 **	7; 5099	1836.24 **	69.08 **	7; 5099	19,950.40 **	42.38 **
Year	4; 12,000	4.64 **	4; 12,000	22.19 **	5.56 **	4; 12,000	46.66 **	6.49 **	4; 12,000	275.18 **	20.92 **
Sex	1; 5085	853.25 **	1; 5099	480.52 **	32.55 **	1; 5099	954.89 **	5.06 *	1; 5099	33,221.30 **	409.77 **
Birth-type–rear-type	2; 5085	3326.84 **	5; 5099	724.59 **	6.36 **	5; 5099	1009.60 **	10.87 **	5; 5099	4108.29 **	NS
Dam age	6; 5085	29.83 **	6; 5099	27.64 **	48.07 **	6; 5099	25.46 **	32.02 **	6; 5099	300.10 **	17.62 **
Sire type	2; 5085	268.01 **	2; 5099	1140.22 **	32.16 **	2; 5099	2409.76 **	56.59 **	2; 5099	4686.92 **	49.80 **
Dam breed (sire type)	1; 5085	344.23 **	1; 5099	719.81 **	67.02 **	1; 5099	1363.61 **	NS	1; 5099	8350.83 **	NS
Sire by year	27; 5075	42.53 **	27; 5099	167.95 **	193.97 **	27; 5099	128.92 **	53.17 **	27; 5099	80.71 **	100.77 **
Site by sire type	NS ^3^	NS	14; 5099	27.16 **	39.86 **	14; 5099	15.67 **	NS	14; 5099	11.70 **	13.56 **
Year by sire type	NS	NS	8; 5099	NS	51.75 **	NS	NS	NS	NS	NS	36.57 **

^1^ NDF: numerator degrees of freedom; ^2^ DDF: denominator degrees of freedom; ^3^ NS: not significant; * *p* < 0.05; ** *p* < 0.01.

**Table 4:** The predicted means of lamb growth for the production factors of lamb sex, lamb birth-type–rear-type, dam age, and dam breed within sire type for actual lamb birth weights and estimated weights and growth rates at 100, 150, and 240 days of age. Within factors, the number of lambs sums to the total number of animals used in the experiment.

	Number of Lambs	Weight (kg)								Growth Rate (g/day)					
		Birth		Day 100		Day 150		Day 240		Day 100		Day 150		Day 240	
		Mean	s.e. ^1^	Mean	s.e.	Mean	s.e.	Mean	s.e.	Mean	s.e.	Mean	s.e.	Mean	s.e.
Sex															
Female	8613	4.54	0.02	26.54	0.13	32.38	0.11	40.58	0.07	136.90	1.84	101.50	2.67	96.44	3.34
Male	8912	4.86	0.02	28.10	0.13	34.14	0.11	43.76	0.07	141.00	1.84	105.40	2.67	127.00	3.34
Birth-type–rear-type															
Single–single	5888	5.62	0.02	31.87	0.12	37.47	0.10	45.47	0.07	140.20	1.69	90.53	2.26	NS ^3^	NS
Twin–single	1440	-	0.02	29.41	0.16	35.27	0.13	43.68	0.07	143.70	2.06	97.83	3.48	NS	NS
Twin–twin	8470	4.60	-	26.75	0.12	32.61	0.10	41.76	0.07	135.70	1.72	104.00	2.17	NS	NS
Triplet–single	195	-	0.03	28.33	0.37	34.33	0.30	42.83	0.12	143.10	4.00	104.20	8.49	NS	NS
Triplet–twin	773	-	-	25.12	0.23	31.21	0.19	40.61	0.09	137.60	2.86	110.01	4.84	NS	NS
Triplet–triplet	759	3.88	-	22.45	0.26	28.66	0.22	38.70	0.10	133.40	3.29	114.30	5.22	NS	NS
Dam age															
2	899	4.34	0.05	27.59	0.32	31.78	0.26	38.90	0.11	118.20	3.71	58.48	6.39	137.50	8.07
3	3525	4.57	0.02	27.40	0.16	33.33	0.13	42.52	0.07	136.70	2.05	100.90	3.12	116.50	3.93
4	4281	4.77	0.02	27.68	0.14	33.98	0.12	42.97	0.07	148.10	1.92	109.50	2.84	107.90	3.64
5	4636	4.78	0.02	27.98	0.14	33.76	0.12	42.63	0.07	138.10	1.93	99.68	2.83	118.40	3.65
6	2739	4.80	0.02	28.64	0.16	33.69	0.13	42.43	0.07	124.50	2.12	78.51	3.35	137.00	4.24
7	1148	4.79	0.03	26.81	0.22	33.39	0.18	42.75	0.09	144.60	2.63	120.80	4.59	93.84	5.65
8	297	4.84	0.06	25.14	0.36	32.89	0.29	43.01	0.12	162.50	4.09	156.60	8.04	71.03	9.35
Dam breed within sire type															
Maternal–Merino	3501	4.59	0.03	27.01	0.22	33.43	0.18	42.52	0.14	146.50	3.45	114.90	3.68	99.01	6.60
Merino–Merino	6620	4.49	0.02	22.87	0.16	27.95	0.13	34.57	0.10	122.60	2.42	84.09	3.01	76.89	4.51
Terminal–Merino	3499	4.79	0.02	29.82	0.18	36.02	0.15	47.07	0.10	142.20	2.50	111.50	2.85	159.30	4.28
Terminal–BLM ^2^	3905	5.25	0.02	34.34	0.17	40.76	0.14	51.79	0.09	153.20	2.47	111.50	2.85	159.30	4.28

^1^ s.e.: standard error; ^2^ BLM: Border Leicester-Merino; ^3^ NS: not significant.

**Table 5:** The range of effect (the difference between the smallest and largest predicted mean) of production factors and of Australian sheep breeding values for sire birth weight (BWT_BV_), weaning weight (WWT_BV_), post-weaning weight (PWT_BV_), fat depth (PFAT_BV_), and eye muscle depth (PEMD_BV_) on lamb weight (kg) and growth rate (g/day), for actual lamb birth weights and estimated weights and growth rates at 100, 150, and 240 days of age.

	Weight (kg)				Growth Rate (g/day)		
	Birth	Day 100	Day 150	Day 240	Day 100	Day 150	Day 240
Site	1.19	10.26	13.20	17.90	79.85	88.63	74.52
Year	0.12	2.72	2.79	1.95	12.16	14.28	88.72
Sex	0.32	1.56	1.76	3.18	4.10	3.90	30.56
Birth-type–rear-type	1.74	9.42	8.81	6.77	10.30	23.77	NS
Dam age	0.50	3.50	2.20	4.11	44.30	98.12	66.47
Sire type	0.53	9.21	10.44	14.86	25.10	30.81	82.41
Dam breed within sire type	0.46	4.52	4.74	4.72	11.00	NS	NS
BWT_BV_	0.95	nm	nm	nm	nm	nm	nm
WWT_BV_	nm ^1^	5.98	nm	nm	NS	nm	nm
PWT_BV_	nm	nm	6.62	8.24	nm	NS	NS
PFAT_BV_	nm	nm	1.41	NS ^2^	nm	NS	NS
PEMD_BV_	nm	nm	NS	NS	nm	NS	NS

^1^ nm: not modelled; ^2^ NS: not significant.

**Table 6:** The regression coefficients for the relationship between production factors and sire Australian sheep breeding values (ASBVs), for sire birth weight (BWT_BV_), weaning weights (WWT_BV_), and post-weaning weight (PWT_BV_) for lamb weight (kg). Coefficients within columns are additive.

	Birth		Day 100		Day 150		Day 240	
	Coefficient	s.e. ^1^	Coefficient	s.e.	Coefficient	s.e.	Coefficient	s.e.
BWT_BV_		WWT_BV_		PWT_BV_		PWT_BV_		
Sire ASBV	0.59	0.09	0.19	0.12	0.17	0.05	0.45	0.03
ASBV * Site								
Kirby			−0.35	0.08	−0.13	0.04		
Trangie			0.07	0.12	0.10	0.06		
Cowra			0.05	0.10	0.09	0.05		
Rutherglen			−0.16	0.09	−0.03	0.04		
Hamilton			−0.26	0.09	−0.13	0.05		
Struan			−0.22	0.10	−0.09	0.05		
Turretfield			−0.09	0.10	−0.03	0.04		
Katanning			0.00		0.00			
ASBV * Year								
2007	−0.27	0.07					−0.21	0.02
2008	−0.22	0.07					−0.05	0.02
2009	−0.30	0.08					−0.05	0.02
2010	−0.33	0.07					−0.03	0.02
2011	0.00						0.00	
ASBV * Birth-type–rear-type								
Single–single	0.33	0.09	0.46	0.08	0.28	0.04		
Twin–single	0.15	0.09	0.39	0.09	0.21	0.04		
Twin–twin			0.23	0.08	0.15	0.04		
Triplet–single	0.00		0.16	0.14	0.11	0.07		
Triplet–twin			0.12	0.11	0.10	0.05		
Triplet–triplet			0.00		0.00			
ASBV * Dam age								
2							−0.07	0.02
3							−0.02	0.02
4							−0.01	0.02
5							−0.01	0.02
6							−0.05	0.02
7							−0.03	0.02
8							0.00	
ASBV * Dam breed within sire type								
Maternal–Merino			−0.01	0.13	−0.06	0.05		
Merino–Merino			0.26	0.09	0.06	0.04		
Terminal–Merino			0.00		0.00			
Terminal–BLM ^2^			0.00		0.00			

^1^ s.e.: standard error; ^2^ BLM: Border Leicester-Merino; * indicates an interaction of terms.

## Data Availability

Data supporting reported results can be requested from the Australian Cooperative Research Centre for Sheep Industry Innovation.
